# New Species of *Talaromyces* (Trichocomaceae, Eurotiales) from Southwestern China

**DOI:** 10.3390/jof8070647

**Published:** 2022-06-21

**Authors:** Xin-Cun Wang, Wen-Ying Zhuang

**Affiliations:** State Key Laboratory of Mycology, Institute of Microbiology, Chinese Academy of Sciences, Beijing 100101, China

**Keywords:** DNA barcodes, fungal biodiversity, phylogeny, taxonomy

## Abstract

Species of *Talaromyces* are cosmopolitan and ubiquitous, and some are of industrial and medicinal importance. Species of *Talaromyces* have been successively reported in China. During our examinations of samples collected from southwestern China, two new species belonging to *Talaromyces* sect. *Talaromyces* were further discovered based on phylogenetic analyses and morphological comparisons. *Talaromyces ginkgonis* sp. nov., isolated from a partially colonized fruit of *Ginkgo biloba*, differs from closely-related fungi in the combination of conidia ellipsoidal, smooth and 3.5−4 × 2−3 μm, no growth on CYA at 37 °C and sequence divergences; *T. shilinensis* sp. nov. is distinguished from its related allies in the combination of smooth conidia, colonies 10−11 mm diameter on CYA at 25 °C and sequence differences. Detailed descriptions and illustrations of the new taxa are given.

## 1. Introduction

Species of *Talaromyces* C.R. Benj. are cosmopolitan and ubiquitous, inhabiting soil, air, indoor environments, rotten food, plant debris, healthy plant as endophytes, insects, and immunodeficient humans. The beneficial and the harmful effects of *Talaromyces* have been well documented [1].

Seven sections have been established and widely accepted in the genus *Talaromyces*: *Bacillispori*, *Helici*, *Islandici*, *Purpurei*, *Subinflati*, *Talaromyces*, and *Trachyspermi* [2,3]. A novel section was recently proposed as sect. *Tenues* [4]. A total of 171 species were compiled in the genus and listed in the latest monograph [3]. Furthermore, 26 new taxa were afterwards noted [1,4,5,6,7,8,9,10,11,12]. Twenty of them are from Asia: *T. albisclerotius* B.D. Sun et al., *T. aspriconidius* B.D. Sun et al., *T. aureolinus* L. Wang, *T. bannicus* L. Wang, *T. brevis* B.D. Sun et al., *T. chongqingensis* X.C. Wang & W.Y. Zhuang, *T. guizhouensis* B.D. Sun et al., *T. gwangjuensis* Hyang B. Lee & T.T.T. Nguyen, *T. haitouensis* L. Wang, *T. koreana* Hyang B. Lee, *T. nanjingensis* X.R. Sun et al., *T. penicillioides* L. Wang, *T. rosarhiza* H. Zhang & Y.L. Jiang, *T. rufus* B.D. Sun et al., *T. sparsus* L. Wang, *T. teleomorpha* Hyang B. Lee et al., *T. tenuis* B.D. Sun et al., *T. wushanicus* X.C. Wang & W.Y. Zhuang, *T. yunnanensis* Doilom & C.F. Liao, and *T. zhenhaiensis* L. Wang; five from Europe: *T. calidominioluteus* Houbraken & Pyrri, *T. gaditanus* (C. Ramírez & A.T. Martínez) Houbraken & Soccio, *T. germanicus* Houbraken & Pyrri, *T. pulveris* Crous, and *T. samsonii* (Quintan.) Houbraken & Pyrri; and one from Africa, *T. africanus* Houbraken et al. *Talaromyces* sect. *Talaromyces* is the largest section and now with 84 species included.

Southwestern China shows various climates, altitudes, and vegetations, and it is rich in fungal biodiversity. Two species from soil in Chongqing were just described [1]. Along with more samples isolated from the area being examined, two additional new species belonging to *Talaromyces* sect. *Talaromyces* were further discovered based on phylogenetic analyses and morphological comparisons. Detailed descriptions and illustrations of the new taxa are provided.

## 2. Materials and Methods

### 2.1. Fungal Materials

The new species were associated with fungal (*Pseudocosmospora* sp.) or plant (*Ginkgo biloba* L.) materials collected in southwestern China (Sichuan and Yunnan provinces) during 2016–2017. Dried cultures were deposited in the Herbarium Mycologicum Academiae Sinicae (HMAS, Beijing, China), and the living ex-type strains were preserved in the China General Microbiological Culture Collection Center (CGMCC, Beijing, China).

### 2.2. Morphological Observations

Morphological characterization was conducted following standardized methods [13]. Four standard growth media were used: Czapek yeast autolysate agar (CYA, yeast extract Oxoid, Hampshire, UK), malt extract agar (MEA, Amresco, Solon, OH, USA), yeast extract agar (YES), and potato dextrose agar (PDA). The methods for inoculation, incubation, microscopic examinations, and digital recordings followed our previous studies [1,14,15,16].

### 2.3. Molecular Experiments

DNA was extracted from the cultures grown on PDA for 7 days, using the Plant Genomic DNA Kit (DP305, TIANGEN Biotech, Beijing, China). Polymerase chain reaction (PCR) amplifications of the internal transcribed spacer (ITS), beta-tubulin (BenA), calmodulin (CaM), and RNA polymerase II second largest subunit (RPB2) gene regions were conducted with routine methods [1,14,15,16]. The products were purified and subjected to sequencing on an ABI 3730 DNA Sequencer (Applied Biosystems, Bedford, MA, USA). Although the ITS region is proposed as the universal DNA barcode for fungi, it is not sufficient to distinguish species of *Talaromyces*. The ITS sequences provided in this study might be helpful for other researchers in case of need.

### 2.4. Phylogenetic Analyses

The forward and the reverse sequences newly generated in this study were assembled using Seqman v. 7.1.0 (DNASTAR Inc., Madison, WI, USA). The assembled sequences were deposited in GenBank. Sequences used for phylogenetic analyses were listed in Table 1. Sequences of each of the three separate datasets (BenA, CaM, and RPB2) and those that were combined were aligned using MAFFT v. 7.221 [17], and then manually edited in BioEdit v. 7.1.10 [18] and MEGA v. 6.0.6 [19]. Maximum Likelihood (ML) analyses were determined using RAxML-HPC2 [20] on XSEDE 8.2.12 on CIPRES Science Gateway v. 3.3 [21] with the default GTRCAT model. Bayesian Inference (BI) analyses were performed with MrBayes v. 3.2.5 [22]. Appropriate nucleotide substitution models and parameters were determined by Modeltest v. 3.7 [23]. The consensus trees were viewed in FigTree v. 1.3.1 (http://tree.bio.ed.ac.uk/software/%20figtree/ accessed on 1 September 2015). The type species *T. trachyspermus* of *Talaromyces* sect. *Trachyspermi* served as an outgroup.

## 3. Results

### 3.1. Phylogenetic Analysis

To infer the phylogeny of *Talaromyces* sect. *Talaromyces* and to determine the positions of the new species, three separate datasets (BenA, CaM and RPB2) and those that were combined were compiled and analyzed. Detailed characteristics of the datasets are listed in Table 2.

**Figure 1:**
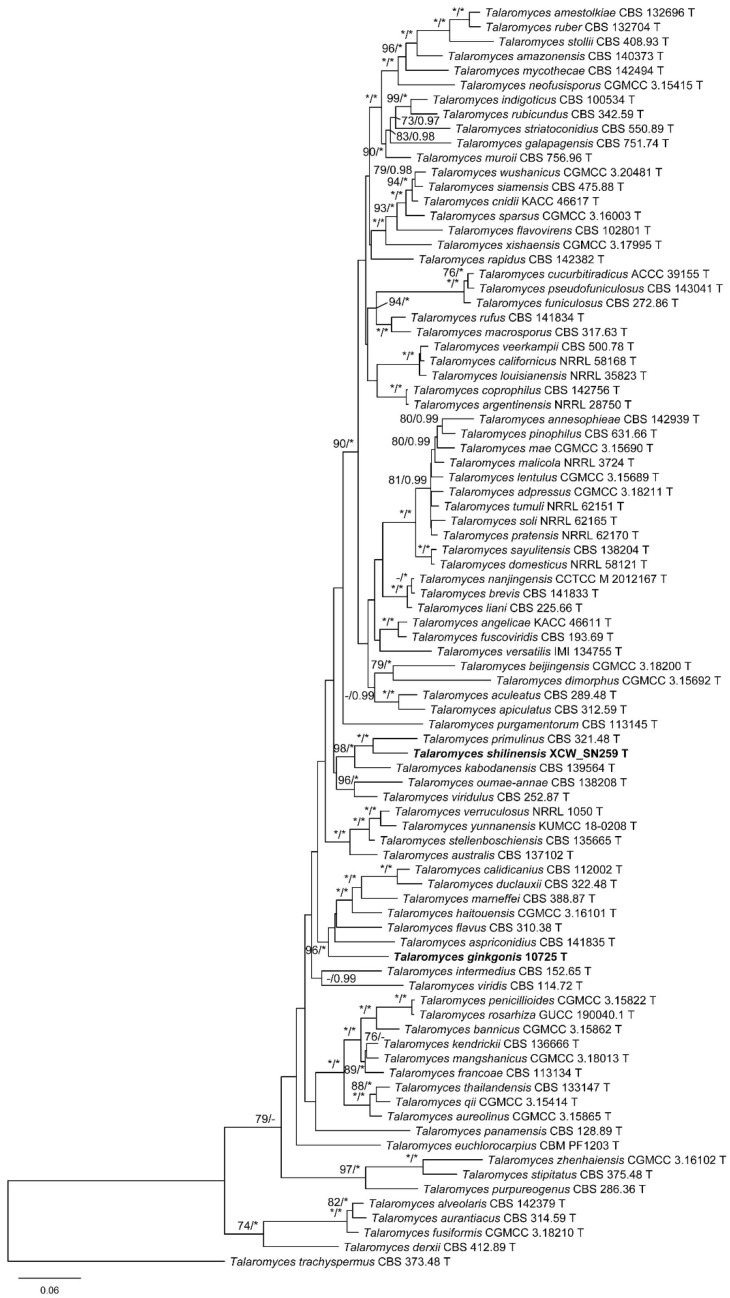
ML phylogeny of *Talaromyces* sect. *Talaromyces* inferred from the combined (BenA + CaM + RPB2) dataset. Bootstrap values ≥70% (**left**) or posterior probability values ≥0.95 (**right**) are indicated at nodes. Asterisk denotes 100% bootstrap or 1.00 posterior probability.

In the BenA phylogeny (Figure S1), the strain 10725 was clustered with *T. aspriconidius*, *T. calidicanius*, *T. duclauxii*, *T. flavus*, *T. haitouensis*, and *T. marneffei*; and XCW_SN259 was grouped with *T. kabodanensis* and *T. primulinus*. In the CaM tree (Figure S2), 10725 showed as a distinct lineage, while XCW_SN259 was a sister taxon of *T. primulinus*. In the RPB2 phylogeny (Figure S3), the position of 10725 was similar to that shown in the BenA phylogeny with relatively weak supports, while the sister relationship between *T. primulinus* and XCW_SN259 was confirmed as that in the CaM phylogeny. In the phylogenetic tree of the combined three-gene dataset (Figure 1), the position of 10725 was identical with the BenA and RPB2 trees and that of XCW_SN259 was consistent in of all the trees (Figure 1 and Figures S1–S3).

### 3.2. Taxonomy

**Figure 2:**
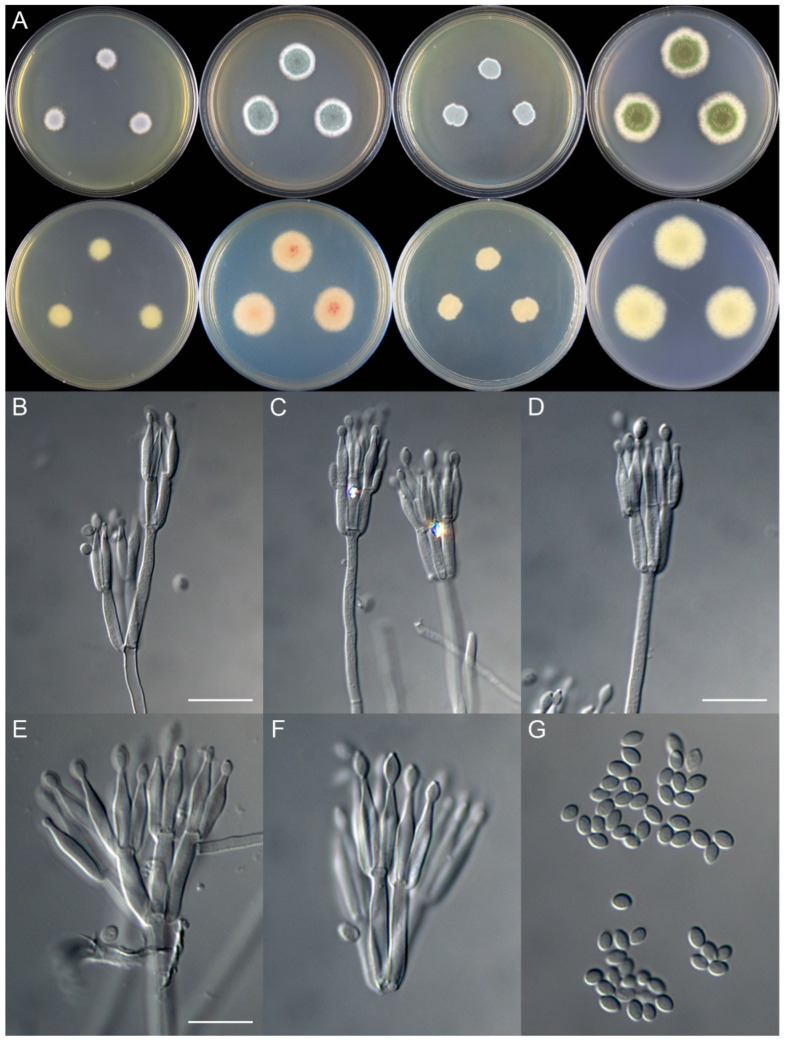
Colonial and microscopic morphology of *Talaromyces ginkgonis* (10725). (**A**) Colony phenotypes (25 °C, 7 days; top row left to right, obverse CYA, MEA, YES, and PDA; bottom row left to right, reverse CYA, MEA, YES, and PDA); (**B**–**F**) Conidiophores; (**G**) Conidia. Bars: B = 15 µm, applies to C; D = 12.5 µm; E = 10 µm, applies to F and G.

***Talaromyces ginkgonis*** X.C. Wang & W.Y. Zhuang, **sp. nov.** Figure 2

Fungal Names: FN570954

Etymology: The specific epithet refers to the substrate of the fungus

in *Talaromyces* sect. *Talaromyces*

Typification: CHINA. Sichuan Province, Chengdu City, Dujiangyan City, Mount Qingcheng, 30°54′8″ N 103°33′40″ E, on a partially colonized fruit of *Ginkgo biloba* L., 22 August 2016, Xin-Cun Wang 10725, cultured by Xin-Cun Wang (holotype HMAS 247853, ex-type strain CGMCC 3.20698)

DNA barcodes: ITS OL638158, BenA OL689844, CaM OL689846, RPB2 OL689848

Colony diam., 7 days, 25 °C (unless stated otherwise): CYA 9–13 mm; CYA 37 °C no growth; MEA 19–21 mm; YES 12–13 mm; PDA 15–27 mm

Colony characteristics: On CYA 25 °C, 7 days: Colonies nearly circular, plain; margins moderately wide, fimbriate; mycelia colorless; texture velutinous; sporulation moderately dense; conidia *en masse* greyish green; soluble pigments absent; exudates absent; reverse greenish white.

On MEA 25 °C, 7 days: Colonies nearly circular, plain; margins wide, fimbriate; mycelia white; texture velutinous; sporulation dense; conidia *en masse* vivid green; soluble pigments absent; exudates absent; reverse buff but pink at centers and white at margins.

On YES 25 °C, 7 days: Colonies irregular, plain; margins narrow, fimbriate; mycelia white; texture velutinous; sporulation dense; conidia *en masse* bluish green; soluble pigments absent; exudates absent; reverse buff at centers, green at periphery, and white at margins.

On PDA 25 °C, 7 days: Colonies nearly circular to irregular, plain; margins wide, irregular; mycelia white; texture velutinous; sporulation dense; conidia *en masse* yellowish green to vivid green; soluble pigments absent; exudates absent; reverse usually pink at centers, green to buff at periphery, and white at margins.

Micromorphology: Conidiophores biverticillate, rarely terverticillate; stipes smooth-walled, 150–360 × 2.0–3.0 μm; metulae 3–5, 11.0–22.5 × 2.0–3.5 μm; phialides acerose, tapering into very thin neck, 3–5 per metula, 12.0–15.0 × 2.0–3.0 μm; conidia ellipsoidal to fusiform, smooth, 3.5–4.0 × 2.0–3.0 μm

Note: This species is phylogenetically related to *T. aspriconidius*, *T. calidicanius*, *T. duclauxii*, *T. flavus*, *T. haitouensis*, and *T. marneffei*, with strong support in the combined three-gene tree (Figure 1). Morphologically, it differs from *T. aspriconidius* and *T. calidicanius* in the smooth conidia; from *T. marneffei* in the ellipsoidal conidia; and from *T. duclauxii*, *T. flavus*, and *T. haitouensis* in the slower growth rate on MEA and YES at 25 °C (Table 3).

**Figure 3:**
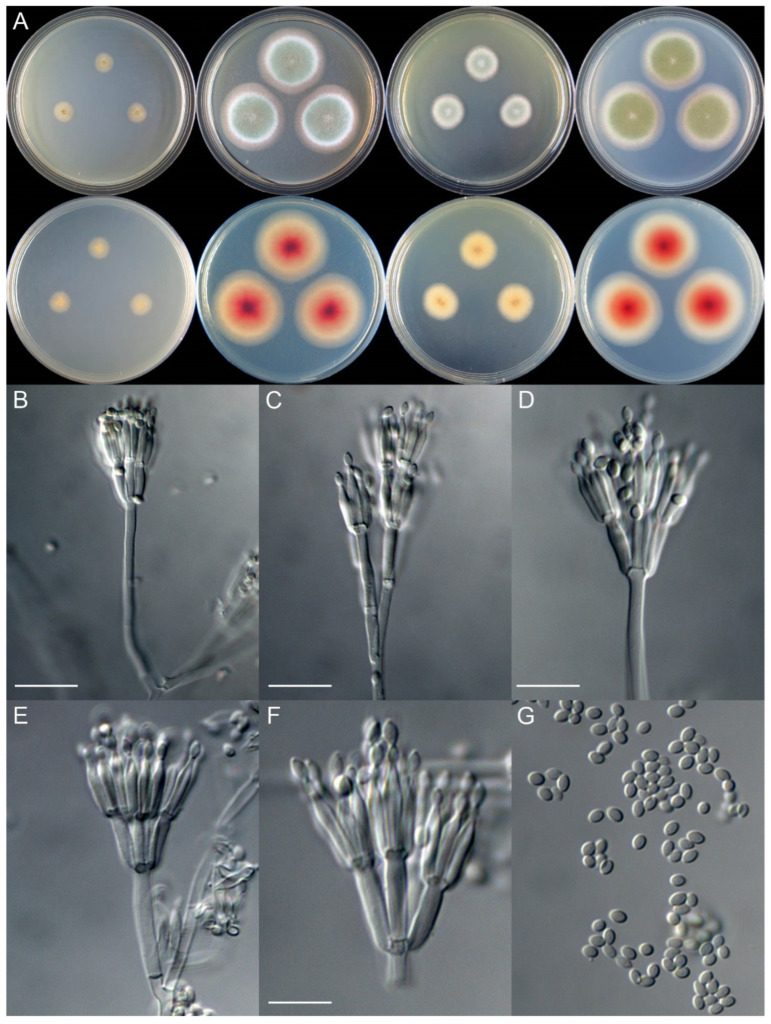
Colonial and microscopic morphology of *Talaromyces shilinensis* (XCW_SN259). (**A**) Colony phenotypes (25 °C, 7 days; top row left to right, obverse CYA, MEA, YES, and PDA; bottom row left to right, reverse CYA, MEA, YES, and PDA); (**B**–**F**) Conidiophores; (**G**) Conidia. Bars: B = 15 µm; C = 12.5 µm; D = 10 µm, applies to E and G; F = 7.5 µm.

***Talaromyces shilinensis*** X.C. Wang & W.Y. Zhuang, **sp. nov.** Figure 3

Fungal Names: FN570955

Etymology: The specific epithet refers to the type locality

in *Talaromyces* sect. *Talaromyces*

Typification: CHINA. Yunnan Province, Kunming City, Shilin Yi Autonomous County, Gui Mountain National Forest Park, 24°38′15″ N 103°35′49″ E, isolated from a rotten twig associated with ascomata of *Pseudocosmospora* sp., 26 September 2017, Yi Zhang, Yu-Bo Zhang and Huan-Di Zheng 11,825, cultured by Yu-Bo Zhang, XCW_SN259 (holotype HMAS 247854, ex-type strain CGMCC 3.20699)

DNA barcodes: ITS OL638159, BenA OL689845, CaM OL689847, RPB2 OL689849

Colony diam., 7 days, 25 °C (unless stated otherwise): CYA 10–11 mm; CYA 37 °C no growth; MEA 36–38 mm; YES 18–19 mm; PDA 35–37 mm

Colony characteristics: On CYA 25 °C, 7 days: Colonies nearly circular, plain; margins wide, entire; mycelia colorless; texture velutinous; sporulation sparse; conidia *en masse* light yellowish green; soluble pigments absent; exudates absent; reverse almost colorless but light brown at centers

On MEA 25 °C, 7 days: Colonies nearly circular, plain, slightly protuberant at centers; margins very wide, entire; mycelia colorless and white; texture velutinous, funiculose at central areas; sporulation dense; conidia *en masse* dull green; soluble pigments absent; exudates absent; reverse buff but pink to reddish brown at centers.

On YES 25 °C, 7 days: Colonies nearly circular, plain, slightly protuberant at centers; margins moderately wide, entire; mycelia colorless; texture velutinous; sporulation dense; conidia *en masse* greyish green; soluble pigments absent; exudates absent; reverse buff but light brown at centers.

On PDA 25 °C, 7 days: Colonies nearly circular, plain, slightly protuberant at centers; margins very wide, entire; mycelia colorless; texture velutinous; sporulation dense; conidia *en masse* dull green; soluble pigments absent; exudates absent; reverse white, pink to reddish brown at centers.

Micromorphology: Conidiophores biverticillate, rarely quaterverticillate; stipes smooth-walled, 50–110 × 2.0–3.0 μm; metulae 4–6, 8.5–12.5 × 2.5–3.0 μm; phialides acerose, tapering into very thin neck, 4–5 per metula, 9.0–13.0 × 1.8–2.5 μm; conidia ellipsoidal to broad-fusiform, smooth, 2.5–3.5 × 2.0–2.5 μm

Note: This species is a sister of *T. primulinus* with strong support in the phylogenies inferred from all datasets (Figure 1 and Figures S1–S3), and it also related to *T. kabodanensis* in the BenA and combined trees (Figure 1 and Figure S1). It has 27 pairwise nucleotide differences from *T. primulinus* and 23 bp from *T. kabodanensis* in the BenA dataset; 29 nucleotide differences from *T. primulinus* in CaM; and 45 nucleotide differences from *T. primulinus* in RPB2. Morphologically, it differs from *T. kabodanensis* in the smooth conidia and from *T. primulinus* in the faster growth rate on CYA, MEA, and YES at 25 °C (Table 3).

## 4. Discussion

Forty-three species of the *Talaromyces* have been reported as new to science based on materials collected from China. They are distributed all over the country, especially in southwestern regions, for example, *T. chongqingensis* and *T. wushanicus* are from Chongqing, *T. albisclerotius*, *T. guizhouensis*, *T. penicillioides*, *T. resinae*, *T. rosarhiza*, and *T. tenuis* are from Guizhou, *T. ginkgonis* is from Sichuan, *T. neofusisporus* and *T. qii* are from Tibet, and *T. aspriconidius*, *T. aureolinus*, *T. bannicus*, *T. rufus*, *T. shiliensis*, and *T. yunnanensis* are from Yunnan [1,4,6,9,10,11]. This proves that southwestern China is one of the global biodiversity hotspots. In northern China, 13 species were recorded from Beijing; in eastern parts of the country, 9 were from Jiangsu, Shandong, Shanghai, Taiwan, and Zhejiang; and a few species were occasionally found in the south, central, and northeast. This might be due to the frequency of investigations, climates, richness of plants, as well as human activities. We certainly expect to discover more species of the group in unexplored regions and even in surveyed areas in different seasons.

Along with the joining of the two new species, *Talaromyces* sect. *Talaromyces* currently possesses up to 86 species around the world. Forty species were originally described as being from Asia, of which 29 are from China, four are from Japan, two are from South Korea and Thailand, respectively, and only one was reported in India, Iran, and Vietnam; 18 taxa are from North America, including 14 from the USA and a single one from Canada, Cuba, Mexico, and Panama; 12 species are distributed in Europe (France, Italy, Netherlands, Spain, UK); six are reported in South America (Brazil, Colombia, and Ecuador); five are from Africa (Ghana and South Africa); and four are from Oceania (Australia and New Zealand). Concerning the known distribution of the genus, one may easily imagine that the biodiversity of *Talaromyces* may have been underrated, although it is well recognized in areas of East Asia and North America, intensive excursions covering a broad range of areas in the world should be suggested to have a better understanding of the biodiversity of this group.

## Figures and Tables

**Table 1:** Fungal species and sequences used in phylogenetic analyses of *Talaromyces* sect. *Talaromyces*.

Species	Strain	Locality	Substrate	ITS	BenA	CaM	RPB2
*T. aculeatus* (Raper & Fennell) Samson et al., 2011	CBS 289.48 T	USA	textile	KF741995	KF741929	KF741975	MH793099
*T. adpressus* A.J. Chen et al., 2016	CGMCC 3.18211 T	China: Beijing	indoor air	KU866657	KU866844	KU866741	KU867001
*T. alveolaris* Guevara-Suarez et al., 2017	CBS 142379 T	USA	human bronchoalveolar lavage	LT558969	LT559086	LT795596	LT795597
*T. amazonensis* N. Yilmaz et al., 2016	CBS 140373 T	Colombia	leaf litter	KX011509	KX011490	KX011502	MN969186
*T. amestolkiae* N. Yilmaz et al., 2012	CBS 132696 T	South Africa	house dust	JX315660	JX315623	KF741937	JX315698
*T. angelicae* S.H. Yu et al., 2013	KACC 46611 T	South Korea	dried root of *Angelica gigas*	KF183638	KF183640	KJ885259	KX961275
*T. annesophieae* Houbraken 2017	CBS 142939 T	Netherlands	soil	MF574592	MF590098	MF590104	MN969199
*T. apiculatus* Samson et al., 2011	CBS 312.59 T	Japan	soil	JN899375	KF741916	KF741950	KM023287
*T. argentinensis* Jurjević & S.W. Peterson 2019	NRRL 28750 T	Ghana	soil	MH793045	MH792917	MH792981	MH793108
*T. aspriconidius* B.D. Sun et al., 2020	CBS 141835 T	China: Yunnan	soil	MN864274	MN863343	MN863320	MN863332
*T. aurantiacus* (J.H. Mill. et al.) Samson et al., 2011	CBS 314.59 T	USA	soil	JN899380	KF741917	KF741951	KX961285
*T. aureolinus* L. Wang 2021	CGMCC 3.15865 T	China: Yunnan	soil	MK837953	MK837937	MK837945	MK837961
*T. australis* Visagie et al., 2015	CBS 137102 T	Australia	soil under pasture	KF741991	KF741922	KF741971	KX961284
*T. bannicus* L. Wang 2021	CGMCC 3.15862 T	China: Yunnan	soil	MK837955	MK837939	MK837947	MK837963
*T. beijingensis* A.J. Chen et al., 2016	CGMCC 3.18200 T	China: Beijing	indoor air	KU866649	KU866837	KU866733	KU866993
*T. brevis* B.D. Sun et al., 2020	CBS 141833 T	China: Beijing	soil	MN864269	MN863338	MN863315	MN863328
*T. calidicanius* (J.L. Chen) Samson et al., 2011	CBS 112002 T	China: Taiwan	soil	JN899319	HQ156944	KF741934	KM023311
*T. californicus* Jurjević & S.W. Peterson 2019	NRRL 58168 T	USA	air	MH793056	MH792928	MH792992	MH793119
*T. cnidii* S.H. Yu et al., 2013	KACC 46617 T	South Korea	dried roots of *Cnidium*	KF183639	KF183641	KJ885266	KM023299
*T. coprophilus* Guevara-Suarez et al., 2020	CBS 142756 T	Spain	herbivore dung	LT899794	LT898319	LT899776	LT899812
*T. cucurbitiradicus* L. Su & Y.C. Niu 2018	ACCC 39155 T	China: Beijing	endophyte from root of *Cucurbita moschata*	KY053254	KY053228	KY053246	n.a.
*T. derxii* Takada & Udagawa 1988	CBS 412.89 T	Japan	cultivated soil	JN899327	JX494306	KF741959	KM023282
*T. dimorphus* X.Z. Jiang & L. Wang 2018	CGMCC 3.15692 T	China: Hainan	forest soil	KY007095	KY007111	KY007103	KY112593
*T. domesticus* Jurjević & S.W. Peterson 2019	NRRL 58121 T	USA	floor swab	MH793055	MH792927	MH792991	MH793118
*T. duclauxii* (Delacr.) Samson et al., 2011	CBS 322.48 T	France	canvas	JN899342	JX091384	KF741955	JN121491
*T. euchlorocarpius* Yaguchi et al., 1999	CBM PF1203 T	Japan	soil	AB176617	KJ865733	KJ885271	KM023303
*T. flavovirens* (Durieu & Mont.) Visagie et al., 2012	CBS 102801 T	Spain	unknown	JN899392	JX091376	KF741933	KX961283
*T. flavus* (Klöcker) Stolk & Samson 1972	CBS 310.38 T	New Zealand	unknown	JN899360	JX494302	KF741949	JF417426
*T. francoae* N. Yilmaz et al., 2016	CBS 113134 T	Colombia	leaf litter	KX011510	KX011489	KX011501	MN969188
*T. funiculosus* (Thom) Samson et al., 2011	CBS 272.86 T	India	*Lagenaria vulgaris*	JN899377	MN969408	KF741945	KM023293
*T. fuscoviridis* Visagie et al., 2015	CBS 193.69 T	Netherlands	soil	KF741979	KF741912	KF741942	MN969156
*T. fusiformis* A.J. Chen et al., 2016	CGMCC 3.18210 T	China: Beijing	indoor air	KU866656	KU866843	KU866740	KU867000
*T. galapagensis* Samson & Mahoney 1977	CBS 751.74 T	Ecuador	soil under *Maytenus obovata*	JN899358	JX091388	KF741966	KX961280
***T. ginkgonis*** X.C. Wang & W.Y. Zhuang sp. nov.	10725 T	China: Sichuan	rotten fruit of *Ginkgo biloba*	**OL638158**	**OL689844**	**OL689846**	**OL689848**
*T. haitouensis* L. Wang 2022	CGMCC 3.16101 T	China: Jiangsu	riverside soil	MZ045695	MZ054634	MZ054637	MZ054631
*T. indigoticus* Takada & Udagawa 1993	CBS 100534 T	Japan	soil	JN899331	JX494308	KF741931	KX961278
*T. intermedius* (Apinis) Stolk & Samson 1972	CBS 152.65 T	UK	swamp soil	JN899332	JX091387	KJ885290	KX961282
*T. kabodanensis* Houbraken et al., 2016	CBS 139564 T	Iran	hypersaline soil	KP851981	KP851986	KP851995	MN969190
*T. kendrickii* Visagie et al., 2015	CBS 136666 T	Canada	forest soil	KF741987	KF741921	KF741967	MN969158
*T. lentulus* X.Z. Jiang & L. Wang 2018	CGMCC 3.15689 T	China: Shandong	soil	KY007088	KY007104	KY007096	KY112586
*T. liani* (Kamyschko) N. Yilmaz et al., 2014	CBS 225.66 T	China	soil	JN899395	JX091380	KJ885257	KX961277
*T. louisianensis* Jurjević & S.W. Peterson 2019	NRRL 35823 T	USA	air	MH793052	MH792924	MH792988	MH793115
*T. macrosporus* (Stolk & Samson) Frisvad et al., 1990	CBS 317.63 T	South Africa	apple juice	JN899333	JX091382	KF741952	KM023292
*T. mae* X.Z. Jiang & L. Wang 2018	CGMCC 3.15690 T	China: Shanghai	forest soil	KY007090	KY007106	KY007098	KY112588
*T. malicola* Jurjević & S.W. Peterson 2019	NRRL 3724 T	Italy	rhizosphere of an apple tree	MH909513	MH909406	MH909459	MH909567
*T. mangshanicus* X.C. Wang & W.Y. Zhuang 2016	CGMCC 3.18013 T	China: Hunan	soil	KX447531	KX447530	KX447528	KX447527
*T. marneffei* (Segretain et al.) Samson et al., 2011	CBS 388.87 T	Vietnam	*Rhizomys sinensis*	JN899344	JX091389	KF741958	KM023283
*T. muroii* Yaguchi et al., 1994	CBS 756.96 T	China: Taiwan	soil	MN431394	KJ865727	KJ885274	KX961276
*T. mycothecae* R.N. Barbosa et al., 2018	CBS 142494 T	Brazil	nest of *Melipona scutellaris*	MF278326	LT855561	LT855564	LT855567
*T. nanjingensis* X.R. Sun et al., 2022	CCTCC M 2012167 T	China: Jiangsu	rhizosphere soil of *Pinus massoniana*	MW130720	MW147759	MW147760	MW147762
*T. neofusisporus* L. Wang 2016	CGMCC 3.15415 T	China: Tibet	leaf sample	KP765385	KP765381	KP765383	MN969165
*T. oumae-annae* Visagie et al., 2014	CBS 138208 T	South Africa	house dust	KJ775720	KJ775213	KJ775425	KX961281
*T. panamensis* (Samson et al.) Samson et al., 2011	CBS 128.89 T	Panama	soil	JN899362	HQ156948	KF741936	KM023284
*T. penicillioides* L. Wang 2021	CGMCC 3.15822 T	China: Guizhou	soil	MK837956	MK837940	MK837948	MK837964
*T. pinophilus* (Hedgc.) Samson et al., 2011	CBS 631.66 T	France	PVC	JN899382	JX091381	KF741964	KM023291
*T. pratensis* Jurjević & S.W. Peterson 2019	NRRL 62170 T	USA	effluent of water treatment plant	MH793075	MH792948	MH793012	MH793139
*T. primulinus* (Pitt) Samson et al., 2011	CBS 321.48 T	USA	unknown	JN899317	JX494305	KF741954	KM023294
*T. pseudofuniculosus* Guevara-Suarez et al., 2020	CBS 143041 T	Spain	herbivore dung	LT899796	LT898323	LT899778	LT899814
*T. purgamentorum* N. Yilmaz et al., 2016	CBS 113145 T	Colombia	leaf litter	KX011504	KX011487	KX011500	MN969189
*T. purpureogenus* (Stoll) Samson et al., 2011	CBS 286.36 T	unknown	unknown	JN899372	JX315639	KF741947	JX315709
*T. qii* L. Wang 2016	CGMCC 3.15414 T	China: Tibet	leaf sample	KP765384	KP765380	KP765382	MN969164
*T. rapidus* Guevara-Suarez et al., 2017	CBS 142382 T	USA	human bronchoalveolar lavage	LT558970	LT559087	LT795600	LT795601
*T. rosarhiza* H. Zhang & Y.L. Jiang 2021	GUCC 190040.1 T	China: Guizhou	endophyte of *Rosa roxburghii*	MZ221603	MZ333143	MZ333137	MZ333141
*T. ruber* (Stoll) N. Yilmaz et al., 2012	CBS 132704 T	UK	aircraft fuel tank	JX315662	JX315629	KF741938	JX315700
*T. rubicundus* (J.H. Mill. et al.) Samson et al., 2011	CBS 342.59 T	USA	soil	JN899384	JX494309	KF741956	KM023296
*T. rufus* B.D. Sun et al., 2020	CBS 141834 T	China: Yunnan	soil	MN864272	MN863341	MN863318	MN863331
*T. sayulitensis* Visagie et al., 2014	CBS 138204 T	Mexico	house dust	KJ775713	KJ775206	KJ775422	MN969146
***T. shilinensis*** X.C. Wang & W.Y. Zhuang sp. nov.	XCW_SN259 T	China: Yunnan	associated with *Pseudocosmospora* sp.	**OL638159**	**OL689845**	**OL689847**	**OL689849**
*T. siamensis* (Manoch & C. Ramírez) Samson et al., 2011	CBS 475.88 T	Thailand	forest soil	JN899385	JX091379	KF741960	KM023279
*T. soli* Jurjević & S.W. Peterson 2019	NRRL 62165 T	USA	soil	MH793074	MH792947	MH793011	MH793138
*T. sparsus* L. Wang 2021	CGMCC 3.16003 T	China: Beijing	soil	MT077182	MT083924	MT083925	MT083926
*T. stellenboschiensis* Visagie & K. Jacobs 2015	CBS 135665 T	South Africa	soil	JX091471	JX091605	JX140683	MN969157
*T. stipitatus* (Thom) C.R. Benj. 1955	CBS 375.48 T	USA	rotting wood	JN899348	KM111288	KF741957	KM023280
*T. stollii* N. Yilmaz et al., 2012	CBS 408.93 T	Netherlands	AIDS patient	JX315674	JX315633	JX315646	JX315712
*T. striatoconidium* (R.F. Castañeda & W. Gams) Houbraken et al., 2020	CBS 550.89 T	Cuba	leaf litter of *Pachyanthus poirettii*	MN431418	MN969441	MN969360	MT156347
*T. thailandensis* Manoch et al., 2013	CBS 133147 T	Thailand	forest soil	JX898041	JX494294	KF741940	KM023307
*T. tumuli* Jurjević & S.W. Peterson 2019	NRRL 62151 T	USA	soil from prairie	MH793071	MH792944	MH793008	MH793135
*T. veerkampii* Visagie et al., 2015	CBS 500.78 T	Columbia	soil	KF741984	KF741918	KF741961	KX961279
*T. verruculosus* (Peyronel) Samson et al., 2011	NRRL 1050 T	USA	soil	KF741994	KF741928	KF741944	KM023306
*T. versatilis* Bridge & Buddie 2013	IMI 134755 T	UK	unknown	MN431395	MN969412	MN969319	MN969161
*T. viridis* (Stolk & G.F. Orr) Arx 1987	CBS 114.72 T	Australia	soil	AF285782	JX494310	KF741935	JN121430
*T. viridulus* Samson et al., 2011	CBS 252.87 T	Australia	soil	JN899314	JX091385	KF741943	JF417422
*T. wushanicus* X.C. Wang & W.Y. Zhuang 2021	CGMCC 3.20481 T	China: Chongqing	soil	MZ356356	MZ361347	MZ361354	MZ361361
*T. xishaensis* X.C. Wang et al., 2016	CGMCC 3.17995 T	China: Hainan	soil	KU644580	KU644581	KU644582	MZ361364
*T. yunnanensis* Doilom & C.F. Liao 2020	KUMCC 18-0208 T	China: Yunnan	rhizosphere soil	MT152339	MT161683	MT178251	n.a.
*T. zhenhaiensis* L. Wang 2022	CGMCC 3.16102 T	China: Zhejiang	mudflat soil	MZ045697	MZ054636	MZ054639	MZ054633
*T. trachyspermus* (Shear) Stolk & Samson 1973	CBS 373.48 T	USA	unknown	JN899354	KF114803	KJ885281	JF417432

GenBank accession numbers in bold indicating the newly generated sequences. Full names of the culture collection centers: ACCC (Agricultural Culture Collection of China); CBS (Centraalbureau voor Schimmelcultures, now Westerdijk Fungal Biodiversity Institute); CCTCC (China Center for Type Culture Collection); CGMCC (China General Microbiological Culture Collection); GUCC (Culture Collection at Department of Plant Pathology, Agriculture College, Guizhou University); IMI (CABI Bioscience); KACC (Korean Agricultural Culture Collection); KUMCC (Kunming Institute of Botany Culture Collection); NRRL (USDA-ARS Culture Collection).

**Table 2:** Detailed characteristics of datasets of *Talaromyces* sect. *Talaromyces*.

Gene Fragment	No. of Seq.	Length of Alignment (bp)	No. of Variable Sites	No. of Parsimony-Informative Sites	Model for BI
BenA	87	643	246	200	K81uf + I + G
CaM	87	581	305	260	SYM + I + G
RPB2	85	978	359	319	TVM + I + G
BenA + CaM + RPB2	87	2202	910	779	GTR + I + G

Full names of the used models: GTR + I + G (General Time Reversible model with unequal base frequencies with Invariable sites and Gamma distribution); K81uf + I + G (Kimura 3-parameter model with unequal base frequencies with Invariable sites and Gamma distribution); SYM + I + G (Symmetrical model with Invariable sites and Gamma distribution); TVM + I + G (Transversion model with Invariable sites and Gamma distribution).

**Table 3:** Morphological comparisons of new *Talaromyces* species and their closely related species.

Species	CYA 25 °C (mm)	CYA 37 °C (mm)	MEA (mm)	YES (mm)	Conidiophore	Conidia Shape	Conidia Wall	Conidia Size (µm)	Reference
*T. aspriconidius*	22–23	22–23	36–37	28–29	biverticillate	globose	strikingly roughened	3–4	[4]
*T. calidicanius*	27–30	no growth	47–48	40–41	biverticillate	ellipsoidal to fusiform	finely rough to rough with spiral striations	2.5–4.5 × 2–3	[2]
*T. duclauxii*	25–27	3–4	48–50	43–44	biverticillate	ellipsoidal	smooth to finely rough	3–4 × 1.5–3.5	[2]
*T. flavus*	9–10	19–20	31–32	24–26	monoverticillate	ellipsoidal	smooth	2–3 × 1.5–2.5	[2]
*T. haitouensis*	22–25	18–20	48–51	25–28	biverticillate	pyriform to ellipsoidal	smooth	2.5–3 × 2–2.5	[10]
*T. marneffei*	13–25	5–10	15–27	17–25	mono- to biverticillate	subglobose	smooth	2.5–4 × 2–3	[2]
*T. ginkgonis*	9–13	no growth	19–21	12–13	biverticillate	ellipsoidal to fusiform	smooth	3.5–4 × 2–3	This study
*T. kabodanensis*	15–25	no growth	37–44	28–35	biverticillate	ovoidal to fusiform	finely rough to rough with spiral striations	2.5–3.5 × 1.5–2.5	[24]
*T. primulinus*	5–6	no growth	20–25	8–10	biverticillate	ellipsoidal to fusiform	smooth to finely rough	2–4 × 1.5–3	[2]
*T. shilinensis*	10–11	no growth	36–38	18–19	biverticillate	ellipsoidal to broad fusiform	smooth	2.5–3.5 × 2–2.5	This study

## Data Availability

The sequences newly generated in this study have been submitted to the GenBank database.

## Supplementary Materials

The following supporting information can be downloaded at: https://www.mdpi.com/article/10.3390/jof8070647/s1, Figure S1: ML phylogeny of *Talaromyces* sect. *Talaromyces* inferred from BenA dataset. Bootstrap values ≥70% (left) or posterior probability values ≥0.95 (right) are indicated at nodes. Asterisk denotes 100% bootstrap or 1.00 posterior probability; Figure S2: ML phylogeny of *Talaromyces* sect. *Talaromyces* inferred from CaM dataset. Bootstrap values ≥70% (left) or posterior probability values ≥0.95 (right) are indicated at nodes. Asterisk denotes 100% bootstrap or 1.00 posterior probability; Figure S3: ML phylogeny of *Talaromyces* sect. *Talaromyces* inferred from RPB2 dataset. Bootstrap values ≥70% (left) or posterior probability values ≥0.95 (right) are indicated at nodes. Asterisk denotes 100% bootstrap or 1.00 posterior probability.

## References

[1] Zhang Z.K., Wang X.C., Zhuang W.Y., Cheng X.H., Zhao P. (2021). New species of *Talaromyces* (Fungi) isolated from soil in southwestern China. Biology.

[2] Yilmaz N., Visagie C.M., Houbraken J., Frisvad J.C., Samson R.A. (2014). Polyphasic taxonomy of the genus *Talaromyces*. Stud. Mycol..

[3] Houbraken J., Kocsube S., Visagie C.M., Yilmaz N., Wang X.C., Meijer M., Kraak B., Hubka V., Bensch K., Samson R.A. (2020). Classification of *Aspergillus*, *Penicillium*, *Talaromyces* and related genera (Eurotiales): An overview of families, genera, subgenera, sections, series and species. Stud. Mycol..

[4] Sun B.D., Chen A.J., Houbraken J., Frisvad J.C., Wu W.P., Wei H.L., Zhou Y.G., Jiang X.Z., Samson R.A. (2020). New section and species in *Talaromyces*. MycoKeys.

[5] Crous P.W., Cowan D.A., Maggs-Kolling G., Yilmaz N., Larsson E., Angelini C., Brandrud T.E., Dearnaley J.D.W., Dima B., Dovana F. (2020). Fungal Planet description sheets: 1112–1181. Persoonia.

[6] Wei S., Xu X., Wang L. (2021). Four new species of *Talaromyces* section *Talaromyces* discovered in China. Mycologia.

[7] Nguyen T.T.T., Frisvad J.C., Kirk P.M., Lim H.J., Lee H.B. (2021). Discovery and extrolite production of three new species of *Talaromyces* belonging to sections *Helici* and *Purpurei* from freshwater in Korea. J. Fungi.

[8] Pyrri I., Visagie C.M., Soccio P., Houbraken J. (2021). Re-evaluation of the taxonomy of *Talaromyces minioluteus*. J. Fungi.

[9] Zhang H., Wei T.P., Mao Y.T., Ma M.X., Ma K., Shen Y., Zheng M.J., Jia W.Y., Luo M.Y., Zeng Y. (2021). *Ascodesmis rosicola* sp. nov. and *Talaromyces rosarhiza* sp. nov., two endophytes from *Rosa roxburghii* in China. Biodivers. Data J..

[10] Han P.J., Sun J.Q., Wang L. (2022). Two new sexual *Talaromyces* species discovered in estuary soil in China. J. Fungi.

[11] Doilom M., Guo J.W., Phookamsak R., Mortimer P.E., Karunarathna S.C., Dong W., Liao C.F., Yan K., Pem D., Suwannarach N. (2020). Screening of phosphate-solubilizing fungi from air and soil in Yunnan, China: Four novel species in *Aspergillus*, *Gongronella*, *Penicillium*, and *Talaromyces*. Front. Microbiol..

[12] Sun X.R., Xu M.Y., Kong W.L., Wu F., Zhang Y., Xie X.L., Li D.W., Wu X.Q. (2022). Fine identification and classification of a novel beneficial *Talaromyces* fungal species from Masson pine rhizosphere soil. J. Fungi.

[13] Visagie C.M., Houbraken J., Frisvad J.C., Hong S.B., Klaassen C.H., Perrone G., Seifert K.A., Varga J., Yaguchi T., Samson R.A. (2014). Identification and nomenclature of the genus *Penicillium*. Stud. Mycol..

[14] Wang X.C., Chen K., Xia Y.W., Wang L., Li T.H., Zhuang W.Y. (2016). A new species of *Talaromyces* (Trichocomaceae) from the Xisha Islands, Hainan, China. Phytotaxa.

[15] Wang X.C., Chen K., Qin W.T., Zhuang W.Y. (2017). *Talaromyces heiheensis* and *T. mangshanicus*, two new species from China. Mycol. Prog..

[16] Wang X.C., Chen K., Zeng Z.Q., Zhuang W.Y. (2017). Phylogeny and morphological analyses of *Penicillium* section *Sclerotiora* (Fungi) lead to the discovery of five new species. Sci. Rep..

[17] Katoh K., Standley D.M. (2013). MAFFT multiple sequence alignment software version 7: Improvements in performance and usability. Mol. Biol. Evol..

[18] Hall T.A. (1999). BioEdit: A user-friendly biological sequence alignment editor and analysis program for Windows 95/98/NT. Nucleic Acids Symp. Ser..

[19] Tamura K., Stecher G., Peterson D., Filipski A., Kumar S. (2013). MEGA6: Molecular Evolutionary Genetics Analysis version 6.0. Mol. Biol. Evol..

[20] Stamatakis A. (2006). RAxML-VI-HPC: Maximum likelihood-based phylogenetic analyses with thousands of taxa and mixed models. Bioinformatics.

[21] Miller M.A., Pfeiffer W., Schwartz T. Creating the CIPRES Science Gateway for inference of large phylogenetic trees. Proceedings of the Gateway Computing Environments Workshop (GCE).

[22] Ronquist F., Teslenko M., van der Mark P., Ayres D.L., Darling A., Hohna S., Larget B., Liu L., Suchard M.A., Huelsenbeck J.P. (2012). MrBayes 3.2: Efficient Bayesian phylogenetic inference and model choice across a large model space. Syst. Biol..

[23] Posada D., Crandall K.A. (1998). MODELTEST: Testing the model of DNA substitution. Bioinformatics.

[24] Crous P.W., Wingfield M.J., Burgess T.I., Hardy G.E., Crane C., Barrett S., Cano-Lira J.F., Le Roux J.J., Thangavel R., Guarro J. (2016). Fungal Planet description sheets: 469–557. Persoonia.

