# Supplementary material for: New Species of Talaromyces (Trichocomaceae, Eurotiales) from Southwestern China
Source: J Fungi (Basel). 2022 Jun 21;8(7):647. doi: 10.3390/jof8070647 (PMC9319149; doi:10.3390/jof8070647)
Supplement: Supplementary file 1 [file jof-08-00647-s001.zip › jof-1736470-supplementary/Supplementary 2nd/Legends.pdf]

Figure S1. ML phylogeny of *Talaromyces* sect. *Talaromyces* inferred from BenA dataset. Bootstrap values ≥ 70% (left) or posterior probability values ≥ 0.95 (right) are indicated at nodes. Asterisk denotes 100% bootstrap or 1.00 posterior probability.

Figure S2. ML phylogeny of *Talaromyces* sect. *Talaromyces* inferred from CaM dataset. Bootstrap values ≥ 70% (left) or posterior probability values ≥ 0.95 (right) are indicated at nodes. Asterisk denotes 100% bootstrap or 1.00 posterior probability.

Figure S3. ML phylogeny of *Talaromyces* sect. *Talaromyces* inferred from RPB2 dataset. Bootstrap values ≥ 70% (left) or posterior probability values ≥ 0.95 (right) are indicated at nodes. Asterisk denotes 100% bootstrap or 1.00 posterior probability.
