# Supplementary figures and images for: New Species of Talaromyces (Trichocomaceae, Eurotiales) from Southwestern China
Source: J Fungi (Basel). 2022 Jun 21;8(7):647. doi: 10.3390/jof8070647 (PMC9319149; doi:10.3390/jof8070647)

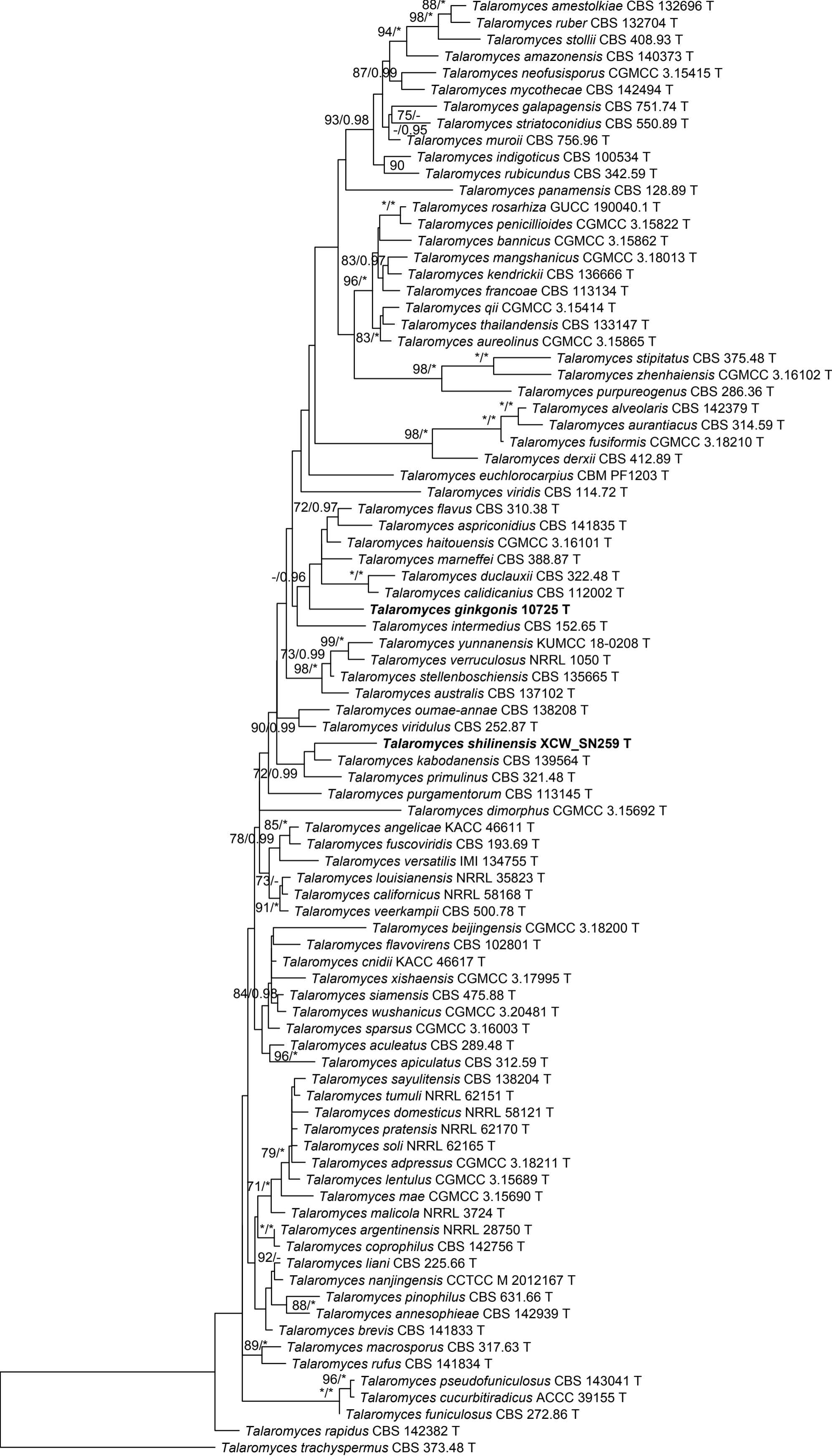

Supplement: Supplementary file 1 [file jof-08-00647-s001.zip › jof-1736470-supplementary/Supplementary 2nd/Figure S1 BenA.pdf]

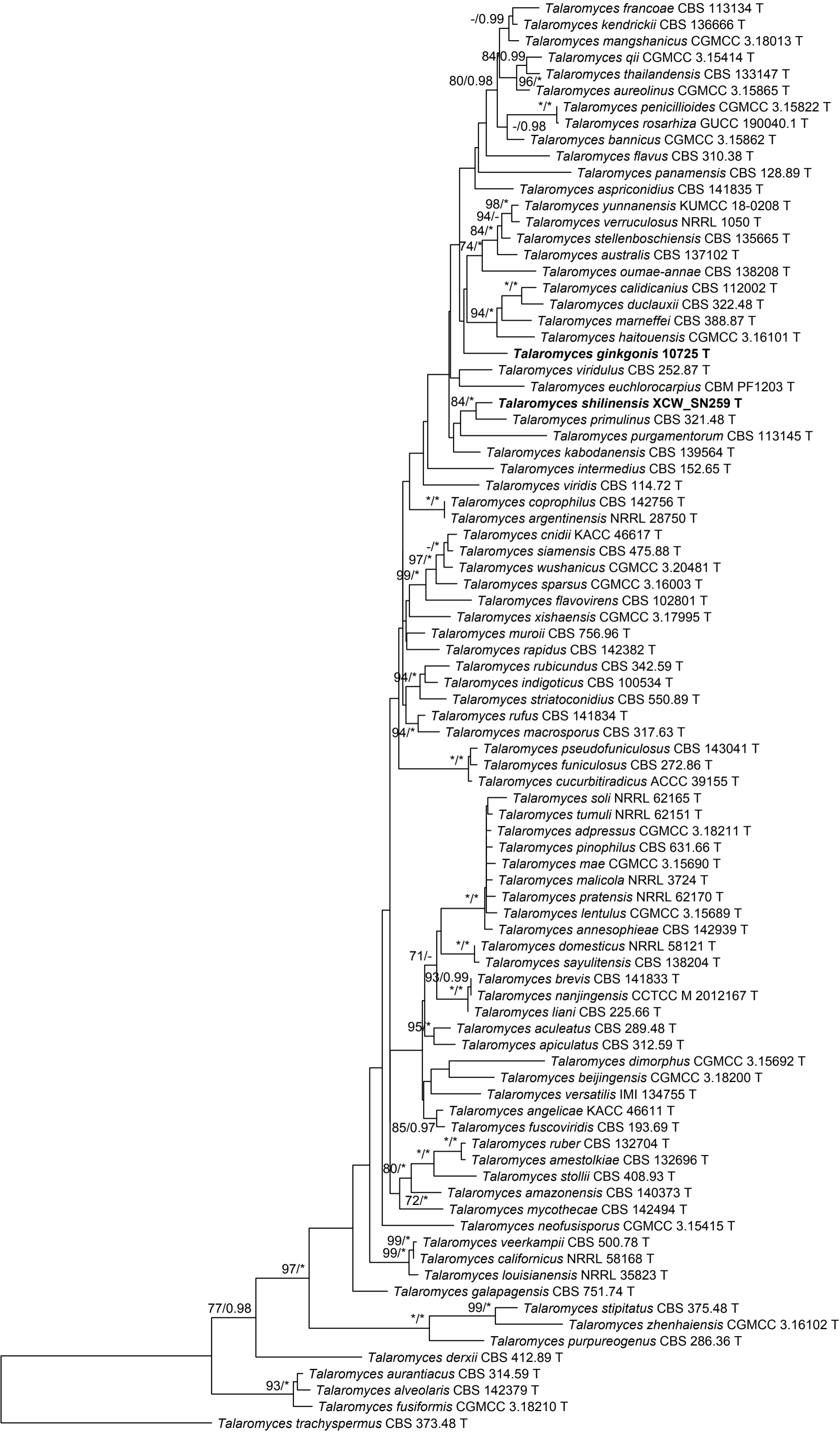

Supplement: Supplementary file 1 [file jof-08-00647-s001.zip › jof-1736470-supplementary/Supplementary 2nd/Figure S2 CaM.pdf]

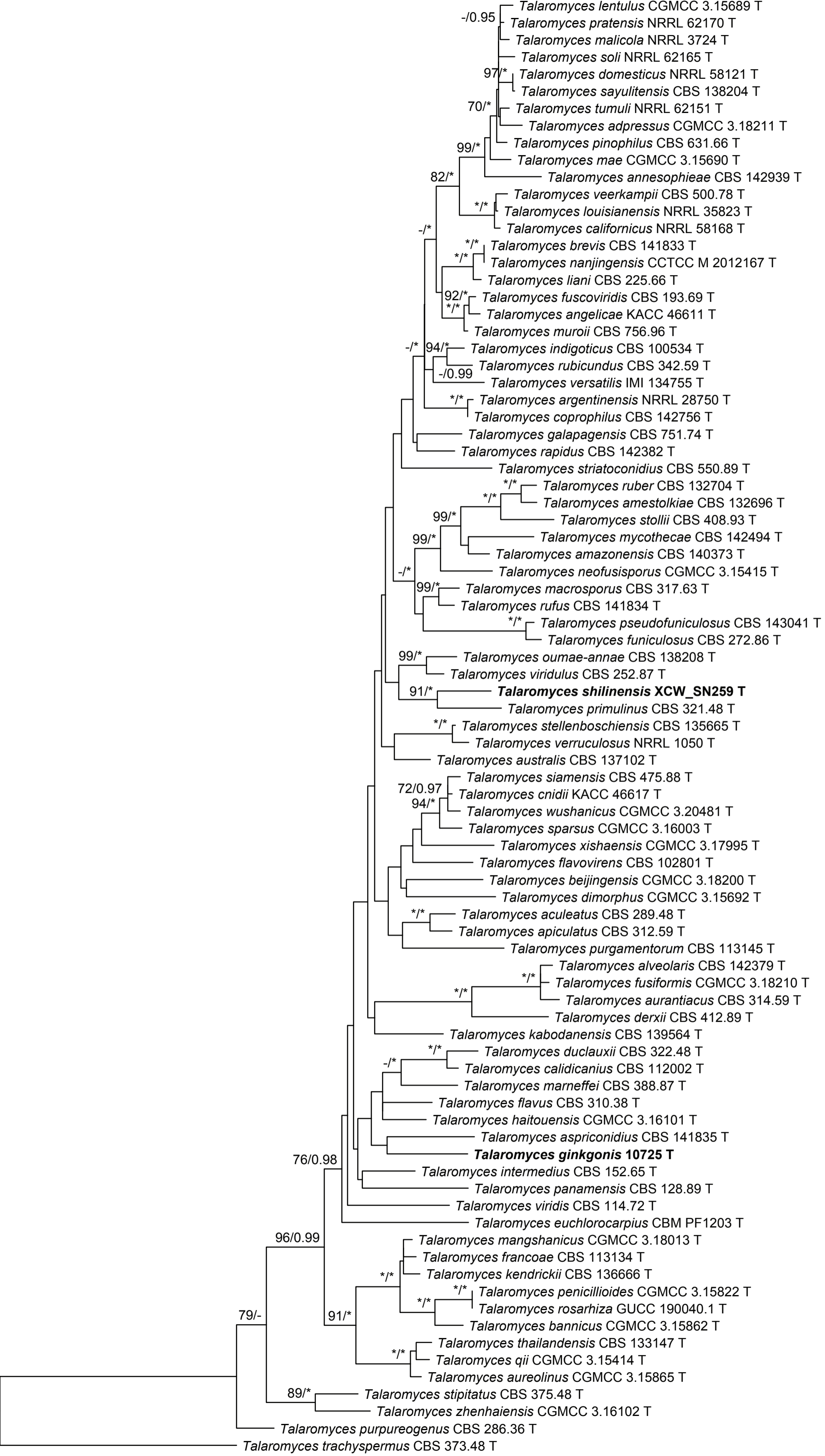

Supplement: Supplementary file 1 [file jof-08-00647-s001.zip › jof-1736470-supplementary/Supplementary 2nd/Figure S3 RPB2.pdf]
